# Acromegaly and non‐parathyroid hormone‐dependent hypercalcemia: a case report and literature review

**DOI:** 10.1186/s12902-021-00756-z

**Published:** 2021-05-01

**Authors:** Shaomin Shi, Lan Zhang, Yerong Yu, Chun Wang, Jianwei Li

**Affiliations:** 1 Department of Endocrinology and Metabolism, West China Hospital, Sichuan University, 610041 Chengdu, China; 2 Department of Endocrinology, Xiangyang Central Hospital, Affiliated Hospital of Hubei University of Arts and Science, Hubei 441000 Xiangyang , China; 3 Department of Endocrinology and Metabolism, Sixth People’s Hospital, First Affiliated Hospital of Chongqing Medical and Pharmaceutical College, 400060 Chongqing, China

**Keywords:** Hypercalcemia, Growth hormone, Prolactin, Acromegaly, Case report

## Abstract

**Background:**

Hypercalcemia associated with acromegaly is mostly parathyroid hormone (PTH)-dependent, being caused by parathyroid hyperplasia or adenoma, which are common in individuals with multiple endocrine adenomatosis-1 (MEN-1). The rare occurrence of non-PTH-dependent hypercalcemia associated with acromegaly is attributable to complex factors involving increased intestinal calcium absorption, enhanced bone calcium release, and reduced urinary calcium elimination. Although patients with acromegaly often have mild hyperphosphatemia and hypercalciuria, clinically significant hypercalcemia is extremely rare.

**Case presentation:**

Here we present a case of non-PTH-dependent hypercalcemia associated with a growth hormone- (GH) and prolactin- (PRL) co-secreting pituitary macroadenoma. A 37-year-old Chinese man presented with a 6-year history of increasing ring and shoe sizes and was referred to the West China Hospital of Sichuan University for treatment of acromegaly. Pituitary magnetic resonance imaging (MRI) showed a 2.0 × 1.7 × 1.9 cm macroadenoma. Laboratory examinations revealed high serum concentrations of GH and PRL with mild hypercalcemia, hyperphosphatemia, hypercalciuria, inhibited PTH concentration, and increased bone turnover markers. Administration of cabergoline together with somatostatin resulted in sharp decreases in his GH, PRL, and serum and urinary calcium concentrations. These values were further reduced 5 months later and his PTH and bone turnover markers gradually returned to within the normal range.

**Conclusions:**

Mild hyperphosphatemia and hypercalciuria are common in individuals with acromegaly and deserve attention because they may contribute to osteoporosis and urolithiasis. However, overt hypercalcemia is rare in such individuals. It is usually attributable to a coexisting parathyroid hyperplasia or adenoma, rarely being non-PTH-dependent. In such cases, the hypercalcemia is attributable to excessive PRL and hypogonadism and reverses with remission of acromegaly.

## Background

Hypercalcemia is reportedly present in approximately 5–10 % of patients with acromegaly [1, 2] and is usually parathyroid hormone (PTH)-dependent as a result of a coexisting parathyroid hyperplasia or adenoma, which are common in individuals with multiple endocrine adenomatosis-1 (MEN-1) [3]. In these patients, hypercalcemia does not usually resolve after elimination of growth hormone (GH) excess, with serum PTH concentrations always being high or at the upper boundary of the normal range. However, there are a few reported cases of non-PTH-dependent hypercalcemia associated with acromegaly. GH and insulin-like growth factor-1 (IGF-1) may increase calcium and phosphate absorption in the intestinal tract and kidney by activating 1-alpha hydroxylase and mediating renal 1,25-dihydroxyvitamin D synthesis [4, 5]. Additionally, enhancement of bone turnover may contribute to hypercalcemia in individuals with acromegaly [6]. It is easy to distinguish between PTH-dependent and non-PTH-dependent hypercalcemia in patients with acromegaly because in the latter, the hypercalcemia is accompanied by inhibition of PTH and is reversed with remission of the acromegaly. Although non-PTH-dependent hypercalcemia in individuals with acromegaly is logical in theory and patients often have mild hyperphosphatemia and hypercalciuria, clinically significant hypercalcemia is rare, possibly because of the strong capacity to self-regulate serum calcium. Here we report a case of non-PTH-dependent hypercalcemia that occurred in a man with acromegaly and discuss potential mechanisms underlying development of hypercalcemia.

## Case presentation

A 37-year-old Chinese man was referred to the West China Hospital of Sichuan University for treatment of acromegaly, having presented with a 6-year history of increasing ring and shoe sizes. Approximately 2 years earlier, he began to experience loss of libido. Other medical history included tuberculous pleurisy that had been successfully treated with anti-tuberculosis drugs 13 years previously and a laparoscopic cholecystectomy for gallstones 5 years previously. No family history of particular disease was present. Over the past 10 years, his body weight had gradually increased by 15 kg. In addition, the patients did not receive vitamin D or vitamin A supplement, and never used any other drugs, including lithium and thiazide. When he first attended our hospital, he was found to be 165 cm tall and weighed 86 kg (BMI 31.59 kg/m^2^). Physical examination revealed a high blood pressure (153/105 mmHg). He exhibited classical features of acromegaly, including enlarged nose, lips, hands, and feet, large pores, and prognathism; his visual field was normal. A review of previous photographs suggested that his appearance had begun to change at least 6 years earlier. Examinations of cardiac, pulmonary, abdominal, and nervous systems were unremarkable.

Pituitary magnetic resonance imaging (MRI) with contrast showed a 2.0 × 1.7 × 1.9-cm sellar tumor that was compressing the pituitary and invading the right cavernous sinus. Laboratory examinations revealed significantly increased serum concentrations of GH (10.97 ng/mL, minimum value after 75 g glucose oral test) and PRL (585 ng/mL, normal range 4.6–21.4 ng/mL), mild hypercalcemia (10.7 mg/dL, normal range 8.5–10.1 mg/dL), hyperphosphatemia (5.48 mg/dL, normal range 2.6–4.6 mg/dL), slightly low PTH concentration (24 pg/mL, normal range 14.5–62.7 pg/mL), and increased bone turnover markers (c-terminal telopeptide [CTX] 1.99 ng/mL, normal range 0.299–0.573 ng/mL; bone alkaline phosphatase [B-ALP] 23.9 ug/L, normal range 11.4–24.6 ug/L). Serum concentrations of testosterone (0.51 ng/mL, normal range 2.5–9.08 ng/mL) were markedly decreased; however, luteinizing hormone (2.5 IU/L, normal range 1.7–8.6 IU/L) and follicle-stimulating hormone (2.2 IU/L, normal range 1.5–12.4 IU/L) were within the normal range, as was thyroid function (Table 1). As to adrenocortical function, on admission his plasma total cortisol (PTC) concentration was 374.1 nmol/L at 08:00. Synchronous adrenocorticotrophic hormone (ACTH) was 43.09 ng/L, whereas post-stress concentrations of PTC and ACTH were 657.3 nmol/L and 45.69 ng/L, respectively. After a 1 mg dexamethasone overnight inhibition test, the serum cortisol concentration at 08:00 was 32.43 nmol/L, indicating that his hypothalamic–pituitary–adrenal axis was functioning normally. Additionally, his liver and renal function, blood lipids, blood glucose, and glycated hemoglobin were all normal. Tumor markers, including alpha-fetoprotein, carcinoembryonic antigen, serum carbohydrate antigen 19 − 9, serum carbohydrate antigen 125, non-small cell lung cancer antigen, neuron specific enolase and total and free prostate specific antigen, were all in the normal range. Chest and abdominal plain and enhanced CT (computerized tomography) showed bilateral pleural thickening and adhesion, no visible gallbladder, and left renal cyst. Ultrasound examination of the genitourinary system showed calcification in the left kidney. Gastroscopy showed mild esophagitis and colonoscopy revealed multiple polyps in rectum and colon, and pathological examination was hyperplastic polyps.

**Table 1:** The patient’s basic parameters and laboratory results

	Normal range	On Admission	2-day after C	1-month after C + S	5-month after C + S
**Bone metabolism**					
Serum Ca	8.5-10.1 mg/dl	10.7	9.5	9.8	9.4
Serum Ph	2.6–4.6 mg/dl	5.48	4.49	3.62	4.15
PTH	14.5-62.7pg/mL	24	38.5	48.7	52.5
Urinary-Ca	100-300 mg/24 h	231.3	-	-	48.6
Urinary-Ph	22-48mmol/24 h	22.33	-	-	15.8
25-OH-D	19-58ng/mL	15.9	-	-	22.8
CTX	0.299-0.573ng/ml	1.99	1.6	0.935	0.660
B-ALP	11.4-24.6ug/L	23.9	20.72	28.78	18.35
Osteocalcin	11-43ng/ml	-	-	-	26.2
**Blood chemistry**					
Serum creatinine	68-108umol/L	61	-	-	71
BUN	3.1-8.0mmol/L	4.4	-	-	4.6
eGFR	56-122ml/min/1.73m^2^	121.2	-	-	113.9
Albumin	40-55 g/L	42	-	-	43
ALT	< 40 IU/L	31	-	-	18
AST	< 40 IU/L	26	-	-	15
Triglyceride	0.29-1.83mmol/L	3.0	-	-	2.71
Cholesterol	2.8-5.7mmol/L	6.12	-	-	5.28
LDL	< 4mmol/L	3.63	-	-	2.88
**Hormones**					
GH-random value	0.03-2.47ng/ml	15.45	3.08	1.14	1.26
OGTT-valley value	< 1ng/ml	10.97	-	-	1.11
IGF-1	86-196ng/ml	> 600	-	193.5	154.85
PRL	4.6-21.4ng/ml	585	92.75	5.51	2.92
T	2.5-9.08ng/ml	0.51	-	-	3.08
LH	1.7–8.6 IU/L	2.5	-	-	2.8
FSH	1.5–12.4 IU/L	2.2	-	-	4.0
PTC(8AM)	147.3-609.3nmol/L	374.1	-	-	337.9
ACTH	5-78ng/L	43.09	-	-	20.05
UFC(24 h)	20.3-127.6ug/24 h	161.8	-	-	112.4
FT3	3.6-7.5pmol/L	5.67	-	-	4.65
FT4	12-22pmol/L	16.51	-	-	17.78
Thyrotropin	0.27-4.2mU/L	0.795	-	-	1.45

*C* indicates cabergoline, *S* somatostatin, *Ca* calcium, *Ph* phosphorus, *PTH* parathyroid hormone, *25-OHD* 25 hydroxyvitamin D, *CTX* type I collagen cross-linked C-telopeptide, *B-ALP* bone-specific alkaline phosphatase, *BUN* blood urea nitrogen, *eGFR* estimated glomerular filtration rate, *ALT* alanine aminotransferase, *AST* aspartate aminotransferase, *LDL* low density lipoprotein, *GH* growth hormone, *OGTT* oral glucose tolerance rest, *IGF-1* insulin-like growth factor-1, *PRL* prolactin, *T* testosterone, *LH* luteinizing hormone, *FSH* follicle-stimulating hormone, *PTC* plasma total cortisone, *ACTH* adrenocorticotrophic hormone, *UFC *urinary free cortisone, *FT3* free triiodothyronine, *FT4* free thyroxine, -, no data

Finally, the patient was diagnosed with a GH and PRL co-secreting pituitary macroadenoma. Surgery was suggested to him, but he refused. So cabergoline together with somatostatin was prescribed (cabergoline 0.5 mg orally twice weekly, octreotide acetate 20 mg intramuscularly every 4 weeks). Thereafter, his GH, PRL, and serum and urinary calcium concentrations decreased sharply, and had reduced further 5 months later. Meanwhile, his PTH and bone turnover markers gradually returned to within the normal range (Table 1). Although the size of the pituitary tumor did not change much after 5 months of follow-up, the patient still refused surgery.

## Literature review

We searched PubMed systematically for reports relating to non-PTH-dependent hypercalcemia associated with acromegaly. The search term was “(Acromegaly OR Growth hormone OR Insulin-like growth hormone-1 OR Prolactin) AND (Hypercalcemia)”. There was no time restriction on the search. The cut-off time for retrieval was November 2020. All reference lists from the main studies and relevant reviews were screened manually for additional eligible studies. Only full-text articles published in English were included. Finally, two articles were identified, including three cases (Table 2), all three of which were from the USA. Biochemical control of disease was achieved in the first patient in Shah et al.‘s study and the patient in Pooja et al.‘s study [1, 4]. The second patient in Shah’s study underwent transsphenoidal pituitary adenoma resection. Her serum calcium concentration normalized within the first 3 postoperative days, but had increased again 2 months later without any medication. The 3 months postoperative MRI scan showed residual tumor, and she failed to achieve biochemical control of disease after subsequent treatment with lanreotide for 5 months followed by fractionated stereotactic radiation therapy for 3 months [1]. The clinical features that all three of these patients with acromegaly and hypercalcemia had in common were as follows: (1) mild hypercalcemia and hypercalciuria, suppressed PTH, and increased 1,25 dihydroxyvitamin D; (2) pathologically diagnosed pituitary adenomas positive for GH and PRL by immunohistochemistry; and (3) changes in serum calcium concentrations paralleled those in GH and IGF-1 concentrations. We did not obtain a pathological diagnosis in the present case but rather diagnosed a GH and PRL co-secreting pituitary macroadenoma based on clinical manifestations and results of investigations.

**Table 2:** The available cases of non-parathyroid hormone-dependent hypercalcemia in acromegaly

Study ID	Age Sex	Course years	Time and reference range	GH ng/ml	IGF-1 ng/ml	PRL ng/ml	Sca mg/dL	Sp mg/dL	Uca mg/24h	PTH Pg/mL	25D ng/ml	1,25D pg/ml
2010Shah1	50/F	2+	on addmission	14.5	911	33	10.3	-	388	20	34	119
			3-month Post	1.4	197	11.3	10.0	-	152	41	48	50
			Normal range	0.03-10	49-292	1-24	8.6-10.2	-	100-300	10-65	30-80	15-75
2010Shah2	51/F	2+	on addmission	75.8	425	67.1	10.7	-	-	19	44	66
			1-week Post	18.5	246	36	8.5	-	-	-	-	-
			2-month Post	12.8	440	10	10.7	-	199	23	30.6	81.3
			Normal range	0.03-10	49-292	1-24	8.6-10.2	-	100-300	10-65	30-80	15-75
2014Pooja	67/F	10+	on addmission	92.0	1498	223.2	10.7	4.1	356.4	13	30.2	72.6
			3-month Post	1.07	304	4.2	9.9	4.3	192.6	25	45.3	38.6
			Normal range	0-3.61	59-225	2-17.4	8.5-10.5	2.5-4.9	100-300	10-60	31-80	15-60
Present report	37/M	6+	on addmission	15.5	>600	585	10.7	5.48	231.3	24	15.9	-
			3-month Post	1.26	154.85	2.92	9.4	4.15	48.6	52.5	22.8	-
			Normal range	0.03-2.5	86-196	4.6-21	8.5-10.1	2.6-4.6	100-300	14-63	19-58	-

*GH* indicates growth hormone, *IGF-1* insulin-like growth factor-1, *PRL* prolactin, *Sca* serum calcium, *Sp* serum phosphorus, *Uca* urinary calcium, *PTH* parathyroid hormone, *25D* 25 hydroxyvitamin D, *1,25D* 1,25-dihydroxyvitamin D, *Post* post-treatment, *F* female, *M* male, -, no data

## Discussion and conclusions

### Mechanisms contributing to the development of hypercalcemia in patients with acromegaly

Hypercalcemia in acromegalics is usually attributable to co-existing primary hyperparathyroidism, which is common in individuals with MEN-1 [7]. Additionally, GH has been shown to stimulate parathyroid hyperplasia in rats [8]; this mechanism may also contribute to the development of parathyroid hyperplasia in humans. However, the present patient’s hypercalcemia was not PTH-dependent. His PTH concentration was close to the lower limit of the normal range with no imaging abnormality in the parathyroid gland, and after the treatment of cabergoline combined with somatostatin, the serum levels of GH, PRL, calcium concentration, urinary calcium concentration, as well as bone turnover markers, were all normalized, with serum PTH increased but not above the normal range. Therefore, mild primary hyperparathyroidism (PHPT) seems unlikely.

Both primary hyperparathyroidism and malignancy are the common causes of hypercalcemia. When the tumors metastasize to the bone or even do not metastasize to the bone, patients with malignant tumors may develop hypercalcemia. Malignancies can produce and release PTH-related peptide (PTHrP) or similar factors, which can mimic the biochemical effects of PTH and cause hypercalcemia. Hypercalcemia caused by PTHrP may present in patients with endocrine neoplasms such as pheochromocytoma, neuroendocrine tumors, and carcinoid tumors, and it can also present in nonendocrine neoplasms [5]. In addition, hypercalcemia has also been reported association with hydrochlorathizide or lithium use which may reduce renal calcium clearance or increase the synthesis of PTH, and with Vitamin A or Vitamin D use which may affect bone absorption and the synthesis of 1,25 dihydroxyvitamin D, and with theophylline use which increases local cyclic AMP levels [5]. However, the present patient’s disease started at age of 37, with no family history and no special medication history. Since ultrasonography showed no abnormality in the parathyroid gland, and the patient’s chest and abdomen CT, cranial MRI, colonoscopy and gastrocopy, and his biochemical examination showed no signs of nodules or tumors. And we excluded MEN-1 and all potential causes of non-PTH-dependent hypercalcemia, such as sarcoidosis, active tuberculosis, and malignancy. The most likely explanation for our patient’s hypercalcemia is that GH inappropriately activated 1-α hydroxylase mediated by IGF-1, which stimulated production of 1,25-dihydroxyvitamin D [9], resulting in increased absorption of calcium in the gut and distal renal tubules [4]. Furthermore, the enhancement of bone turnover mediated by GH may have contributed to his hypercalcemia [6]. However, using radioisotopic calcium, Sigurdsson et al. demonstrated that the major cause of hypercalcemia and hypercalciuria in acromegalics is increased calcium absorption from the gut [10]. Moreover, the normalization of calcium and phosphorus metabolism after biochemical remission of acromegaly is consistent with the above explanation [1, 4, 11, 12].

Although development of non-PTH-dependent hypercalcemia in individuals with acromegaly is theoretically logical, it is rare in clinical practice, possibly because of the strong capacity to self-regulate serum calcium concentrations. Approximately 30–60 % of patients with acromegaly have mild hyperphosphatemia and hypercalciuria, which may contribute to urolithiasis [13, 14]. Although serum calcium concentrations tend to increase [15], they generally remain within the normal range [6]. The rare development of hypercalcemia in acromegaly is reportedly usually attributable to co-existent primary hyperparathyroidism. Constantin et al. prospectively studied calcium and bone turnover markers in 22 patients with acromegaly and compared the findings with those in 22 patients with nonfunctioning pituitary adenomas. Two of their patients with acromegaly (2/22) developed non-PTH dependent hypercalcemia; however, no details concerning these two patients were provided. Whether they were taking vitamin D and/or calcium supplements, or receiving physiologic doses of hydrocortisone was not reported, such treatment may lead to hypercalcemia, as occurred in other patients in that study [6]. Takamoto et al. reported calcium homeostasis in 12 patients with acromegaly treated with pituitary adenomectomy. Only one of these patients, a 47-year-old man, was found to have hypercalcemia with normal PTH and a high serum PRL (100 ng/mL); however, no details were provided [16]. We found only three reported cases of non-PTH-dependent hypercalcemia associated with acromegaly, all of which had pathologically confirmed adenomas that were positive for expression of GH and PRL by immunohistochemistry. Although we did not obtain a pathological diagnosis in the present patient, we diagnosed him as having a GH and PRL co-secreting pituitary macroadenoma based on the clinical manifestations and his response to treatment with a combination of cabergoline and somatostatin. In animals, PRL increases the activity of 1-α hydroxylase [17], and serum 1,25-dihydroxyvitamin D concentrations have been found to be increased in lactating rats [18]; however, the effect of PRL on calcium regulation in humans is still unclear. Serum concentrations of 1,25-dihydroxyvitamin D in patients with prolactinoma are controversial and require further clarification [1]. Meanwhile, no cases of hypercalcemia related to prolactinoma have been reported thus far, other than in individuals with MEN-1. Although the effect of PRL on calcium metabolism may be slight, its effects in combination with GH could overwhelm the capacity to self-regulate serum calcium, resulting in detectable hypercalcemia.

Our patient also had hypogonadism, which is common in individuals with prolactinoma or acromegaly as a consequence of the effects of excess PRL on the hypothalamic–pituitary–gonadal axis or the mass effect of the tumor [13]. GH potentially increases bone turnover, with bone formation and absorption coupled and bone absorption slightly dominant [19]; however, serum calcium concentrations generally remain within the normal range. The presence of hypogonadism may shift the balance toward bone absorption [20], which may disturb calcium homeostasis, contributing to hypercalcemia and eventually an increased risk of vertebral fractures, as documented by others [21].

In summary, the proposed mechanisms for the present patient’s hypercalcemia include: (1) increased calcium absorption in the intestinal tract and kidney, which may be attributable to the combination of 1,25-dihydroxyvitamin D overproduction and enhancement of bone turnover mediated by excess GH; (2) 1,25-dihydroxyvitamin D overproduction potentially mediated by excess PRL; and (3) excessive bone absorption exacerbated by hypogonadism.

### Bone quality in acromegaly

The effects of GH and IGF-1 on bone are complex, including stimulation of osteoblast differentiation, inducing osteoprotegerin production, receptor activator of nuclear factor kappa-B ligand (RANK-L) synthesis and osteoclastogenesis, which may be partially mediated by 1,25-dihydroxyvitamin D [20, 21]. Acromegaly is usually associated with increased bone turnover, which is characterized by coupling of bone formation and resorption with bone absorption slightly dominant and serum calcium balanced. Markers of bone formation and resorption such as osteocalcin, B-ALP, n-terminal telopeptide, and CTX are generally increased [13]. In the present patient, B-ALP and CTX were increased, with CTX dominant, and they gradually normalized after remission of the patient’s acromegaly [6].

Bone mineral density (BMD), as measured by dual energy X-ray absorptiometry (DXA), has been variously reported to be normal, increased, or decreased by different researchers. No matter the BMD according to DXA, the prevalence of morphometric vertebral fractures in acromegalics is higher than in the general population (39–59 % vs. 14 %); this phenomenon persists even after biochemical control of acromegaly [19–21]. BMD according to DXA reportedly does not correlate with fracture risk in patients with acromegaly, whereas the risk of fracture is associated with hypogonadism, long duration of acromegaly, active acromegaly, diabetes mellitus, and glucocorticoid over-replacement, all of which can potentially worsen bone quality [21]. There is controversial evidence that bone microarchitecture rather than bone quantity is impaired; thus, BMD may not be an ideal means of identifying bone damage in acromegalics. Therefore, other means of reflecting bone microarchitecture, such as trabecular bone score and high-resolution peripheral quantitative computed tomography, have been introduced [20, 21] and may be preferable in acromegaly patients.

In summary, mild hyperphosphatemia and hypercalciuria are common in individuals with acromegaly and deserve attention because they may contribute to osteoporosis and urolithiasis. However, overt hypercalcemia is rare in these patients and is not always attributable to coexistent parathyroid hyperplasia or adenoma. It is rarely non-PTH-dependent, may be exacerbated by excess PRL and hypogonadism, and can resolve with remission of acromegaly. Moreover, acromegalics have an increased prevalence of vertebral fractures and BMD may not be the ideal means of assessing bone damage in such patients. Further studies are needed to clarify the development of non-PTH-dependent hypercalcemia and bone damage in individuals with acromegaly.

### Limitations

This study had several limitations. First, we are unable to measure 1,25-dihydroxyvitamin D, and only measured serum 25 hydroxyvitamin D. This was low, which is consistent with past reports and may be partly attributable to excessive conversion of 25 hydroxyvitamin D to 1,25-dihydroxyvitamin D. Second, the present patient’s diagnosis was not pathologically confirmed because he refused surgery. Third, the results included were limited to full-text articles published in English; thus, other reports may exist.

## Data Availability

Data sharing is not applicable to this article because no datasets were generated or analyzed during the current study.

## Abbreviations

ALT: Alanine aminotransferase
AST: Aspartate aminotransferase
ALP: Bone-specific alkaline phosphatase
BMD: Bone mineral density
BUN: Blood urea nitrogen
CTX: Type I collagen cross-linked C-telopeptide
DXA: Dual energy X-ray absorptiometry
eGFR: Estimated glomerular filtration rate
GH: Growth hormone
IGF-1: Insulin-like growth factor-1
LDL: Low density lipoprotein
MRI: Magnetic resonance imaging
PRL: Prolactin
PTH: Parathyroid hormone
25-OHD: 25 hydroxyvitamin D
